# Patients in 24-hour home care striving for control and safety

**DOI:** 10.1186/1472-6955-11-9

**Published:** 2012-06-14

**Authors:** Lena Swedberg, Eva Hammar Chiriac, Lena Törnkvist, Ingrid Hylander

**Affiliations:** 1 Department of Neurobiology, Care Science and Society, Centre for Family Medicine (CeFAM), Karolinska Institutet, Alfred Nobels alle´12, S-14284, Huddinge, Sweden; 2 Department of Behavioural Sciences and Learning, Linköping University, S-581 83, Linköping, Sweden

## Abstract

**Background:**

This article concerns Swedish patients receiving 24-hour home care from health care assistants (HC assistants) employed by the municipality. Home care is a complex interactive process involving the patient, family, HC assistants as well as professional care providers. Previous studies exploring patient perspectives on home care have been based mainly on patient interviews. In contrast, the present study took a broad perspective on patients’ experiences and thoughts by combining field observations on care situations with patient and HC assistant interviews. The aim of the study presented in this article was to promote a new and broadened understanding of patients receiving 24-hour home care by constructing a theoretical model to illuminate their main concern.

**Methods:**

Field observations and semi-structured interviews were conducted with four patients receiving 24-hour home care and their HC assistants. Grounded theory methodology was used.

**Results:**

The core process identified was *Grasping the lifeline*, which describes compensatory processes through which patients strived for control and safe care when experiencing a number of exposed states due to inadequate home care. Patients tried to *take control* by selecting their own HC assistants and *sought safe hands* by instructing untrained HC assistants in care procedures. When *navigating the care system*, the patients maintained contacts with professional care providers and coordinated their own care. When necessary, a devoted HC assistant could take over the navigating role. The results are illuminated in a theoretical model.

**Conclusions:**

The results accentuate the importance to patients of participating in their own care, especially in the selection of HC assistants. The model illustrates some challenging areas for improvement within the organisation of 24-hour home care, such as personnel continuity and competence, collaboration, and routines for acute care. Furthermore, it may be used as a basis for reflection during the planning of care for individual patients within home care.

## Background

The increasing need for home care, both globally and in Sweden, has been described as challenging [1-3]. Today medically fragile individuals, who are often dependent on technology, are cared for at home more frequently than in the past [4,5]. When patients choose home care, they often value it highly, because it increases their chances of living a close-to-normal life despite their considerable need of care [6-8]. However, patients who receive home care due to their large needs may still suffer when they experience the loss of physical functions or changes in self-integrity [9]; such experiences can lead them to develop different strategies to cope with their difficult life situation [10].

The majority of medically fragile and technology-dependent patients are in need of help from employed caregivers around the clock, even if they also practise self-care, receive assistance from family caregivers and have intermittent contacts with health care professionals such as district nurses and physicians [11,12]. Previous studies on patient experience during home care have revealed problems caused by the lack of training and instruction of the caregivers, especially when the care involves the use of technical medical equipment [6-8,13]. When patients require 24-hour care, there are even greater challenges related to competence, a factor that is crucial when evaluating the quality of home care [14,15]. Caregivers working within home care in Sweden seldom have formal education in advanced care procedures [16,17], and, frequently, they are delegated to perform medical and technical tasks by health care professionals [18].

Patients who receive 24-hour home care often have complex needs and thus, depending on the structure of the local organisation for home care. In Sweden the patients often have to rely on collaboration between the local municipality, responsible for the daily care at home, and several actors within primary as well as specialist care with authorizations from the county council [19]. However, the need for collaboration is often insufficiently met, with great regional variation [20], which leads to problems concerning shared information, boundaries of responsibilities, and evaluation methods [12,19]. Lack of collaboration between health care professionals and poor management within organisations for home care may result in extended hospital stays and difficulties in planning care that affect quality of care and of life [20-22].

Home care is a complex interactive process often involving the patient, family, HC assistants as well as professional care providers. Further research is needed on patients’ perception of their own situation, to gain knowledge necessary for the development of future care systems. Whereas earlier studies were based mainly on patient interviews [6,7], the study described herein took a broad perspective: it focused on the patients’ situation by combining field observations on care situations with patient and HC assistant interviews.

The aim of this field study was to gain new and broader understanding of patients receiving 24-hour home care by constructing a theoretical model to illuminate their main concern.

## Methods

### Definitions

In Sweden, the local municipality and the county council are generally co-responsible for home care (Table 1). In the current context, *24-hour home care* was defined as the need for care around the clock by employed caregivers. The care included both *basic care* such as assistance with personal hygiene, mobilization and help at meals, as well as *advanced care* such as specific care procedures including administration of medication, tube-feeding and handling of life-saving technology. End-of-life care was not included. The caregivers, in this study named *health care assistants (HC assistants),* were organised and employed by the local municipality. In addition to providing basic care, HC assistants also performed advanced care delegated by health care professionals. *Professional care provider was* used to collectively name *home care supervisors* at the municipality, and *health care professionals* within primary and specialist care authorised by the county council.

**Table 1:** Home care in Sweden: A joint responsibility between municipality and county council

**Authority**	**Responsibility**	**Care providers**
**Local municipality**	**Home care**	**Health care assistants**
Home health care/ social services	based on patient needs	Primary caregivers
		Managed by supervisors in the municipality
		May perform care procedures delegated by health care professionals
**County council**	**Primary care & specialist care**	**Health care professionals**
Primary care centre & hospital settings	based on patient needs	(i.e. physicians, nurses, physiotherapists) authorized by county council

### Design

The purpose of the study was to improve understanding of patients who are receiving 24-hour home care; thus, a field study was chosen, because it is suitable for exploring a course of events or processes to try to understand, interpret, and describe a specific authentic setting. The unique characteristic of the field study approach is that the researcher collects data in the participants’ natural setting and uses more than one data collection method, one of which is usually observation, to attempt to understand the phenomena in question [23-25].

Grounded theory methodology (GTM) was used to analyse the data because this approach focuses on interactions and social events [26-28] and aims to generate theories and concepts that are grounded in empirical data [26]. Furthermore, it deals with interactions in their natural and daily context, which, in the present study, include the interactions of patients cared for at home with their HC assistants. The type of grounded theory method used was similar to classic grounded theory, in that it emphasised conceptualisation [28]. In its ontology, however, we rely on a constructivist approach [29] and recognise the roots in symbolic interactionism [26]. The approach is outlined by Hylander [27]; for recent examples of its use see Berlin [30] and Modin [31]. The outcome of the study – the theoretical model – was constructed by the researchers, and consists of a set of hypotheses, although these are well grounded in the data.

### Data collection

During 2007–2010, a field study was conducted in the homes of four patients by the lead author. Before the study, a supervisor at the local municipality was approached to help with making the first contacts with patients and their HC assistants. Oral and written information about the study was provided and written consent was obtained. Field observations (total duration 78 hours) and individual interviews were conducted on 17 occasions with the four patients and 12 HC assistants (Table 2).

**Table 2:** Settings and participants

**Setting**	**Hours of observation**	**Interviews**	**Observed**
			**HC assistant**
			(not interviewed)
**Female patient**	20	Patient	3
Age 75		HC assistant (F, age 45)	
Neuromuscular diagnosis			
Immobilised			
**Male patient**	30	Patient	4
Age 25		HC assistant (F, age 42)	
Neurological diagnosis		HC assistant (F, age 24)	
Immobilised		HC assistant (F, age 31)	
Home ventilator		HC assistant (M, age 40)	
**Female patient**	20	Patient	
Age 70		HC assistant (F, age 53)	
Rheumatic diagnosis		HC assistant (F, age 33)	
Immobilised		HC assistant (F, age 40)	
		HC assistant (F, age 20)	
**Male patient**	8	Patient	
Age 62		HC assistant (F, age 47)	
Neurological diagnosis		HC assistant (F, age 26)	
Immobilised		HC assistant (F, age 35)	
**Total.** Settings: **(n) 4**	Hours: **(n) 78**	Interviews:	Observed HC
		Patients **(n) 4**	assistants (n) 7
		HC assistants **(n) 12**	

*The qualitative face-to-face interviews*, which ranged in length from 20 to 60 minutes, followed a semi-structured interview guide with open-ended questions.

The patients were asked to describe experiences and feelings regarding:

Own care needs, decision-making, relation to their HA assistants, contact with professional care providers and areas of improvement.

The HA assistants were asked to describe experiences and feelings regarding:

Patients’ care needs, relation to patient, contact with professional care providers and areas of improvement.

In addition to the planned interviews, short field interviews were conducted and reported in the fieldnotes [32]. Memos were written directly after each interview and during the analyses. The interviews were audio-taped and transcribed verbatim. Each transcript was analysed before the next interview to identify important issues, questions, and ideas about links between emerging codes. The memos were used to modify the interview guide and were a basis for the analysis.

*The field observations* were direct (no time delay between occurrence and registration) and theory-building with a low level of structure for data gathering [29,33]. The lead author visited the patients’ homes frequently over a period of time and participated in small talk to become familiar with the informants, but did not become involved in care situations. The focus of the observations were care situations and patient-HC assistant interactions. The aim of constructing a theoretical model guided the structure of the observations in such a way that the first observations were very open, but they became more focused once theoretical categories and processes had been created. In addition to direct observation notes, field notes were written immediately after the observation.

The authenticity was judged against two criteria: 1) whether a situation would occur irrespective of the researcher’s participation (researcher perspective), and 2) whether the interaction between individuals would exist irrespective of the participants’ awareness of the observation (participant perspective) [34].

### Settings and participants

In accordance with GTM, theoretical sampling was conducted by visiting each patient several times to obtain a range of data and to fill the categories of the emerging model. This allowed observation of variations in the care situations as well as the opportunity to meet several HC assistants.

The *patients* in the study, two females and two males (age range 25–71 years), lived in their own homes in a medium-sized Swedish city. They were totally immobilized and depended on one or two HC assistants being present around the clock (range 8–17 caregivers per patient). Their needs were both basic, such as assistance with personal hygiene, mobilization, and help at meals, and advanced, such as specific care procedures, including administration of medication, tube-feeding, and oxygen, as well as handling life-saving technology and wound care. One patient, who was in need of a home ventilator, had impaired verbal skills due to a tracheotomy and communicated via whispering and nonverbal methods. The other patients could communicate verbally. There were no next-of-kin living with the patients. Twelve *HC assistants* were interviewed and observed during care situations (11 females and one male; age range 20–53 years). They had a variety of educational backgrounds (ranging from licensed nurse qualifications to no health care education at all) and a range of work experience within home care (from a few months to several years). None of the HC assistants were related to the patients. An additional seven HC assistants were observed during care situations (five females and two men) who were not interviewed in the study (Table 2).

### Analysis

Analysis of the data included coding at increasingly abstract levels and constant comparison. The analysis began when the first observations and interviews had been conducted and continued concurrently with data collection. On the basis of the GTM, *open*, *theoretical*, and *selective coding* were performed in order to develop a theoretical model that was well grounded in data. In the *open coding*, transcripts were read line by line and incidences coded; 33 codes were identified, for example *handled like glass* and *hanging loose.* The patients’ and HC assistants’ own words, together with data from the observed actions, were used as much as possible to capture the *substance of data*. Comparisons were made incident-by-incident, similarities and differences were identified, and new codes were condensed into the categories *control* and *exposure*, which had a number of subcategories. In the *theoretical coding*, patterns and connections emerged from the empirical data in response to the question: how do the concepts relate to each other? The main categories developed into main processes, such as *losing the lifeline*, *grasping the lifeline*, and *holding on to the lifeline*. In the *selective coding*, data were coded against the basic social process of the strategies of patients for *grasping the lifeline*. A theoretical model was developed to illuminate the results. Citations, interview quotes from patients (p) and HC assistants (a), as well as observation notes (obs.) were used to illustrate the findings. Given the small sample size, confidentiality was maintained by using the pronoun *she* when quoting the four patients and the HC assistants, regardless of their sex.

### Ethical approval

The study was approved by the Ethical Committee of Huddinge University Hospital, Karolinska Institutet (registration number 2007/170-31). Informed consent was obtained after the participants had been provided with oral and written information about the study. Participant confidentiality was guaranteed, with special consideration given to the patients enrolled in the study. For this reason, the quotations have not been identified. Tapes were destroyed shortly after the transcription process.

## Results

The theoretical model that resulted from the study illustrated that patients in need of 24-hour home care strive for control and safe care (Table 3). The initial analysis revealed a number of *exposed states*, which described patients’ feelings of lack of control, being in unsafe hands and insecure in the care system, and thus experiencing a care system inappropriate to their needs. The core process identified was *Grasping the lifeline*, which describes the compensatory process that patients used to make up for their exposed state. The process termed *grasping the lifeline* included three sub processes that described patient striving: 1) *Taking control*, 2) *seeking safe hands*, and 3) *navigating in the care system*. When patients could be said to be *holding on to the lifeline* their striving showed the desired effect and they felt in control and safe. They could be described as *losing the lifeline* when they gave up their striving or the striving had no effect, and the patient felt a lack of control and in unsafe care. The results of the compensatory processes were influenced by the interaction between the patients and their HC assistants and were different for each individual depending on how the sub processes interacted. This clarified the variation in the data. Furthermore, the theoretical model should be seen as nonlinear and nonstatic because the exposed states, as well as the patients’ abilities to compensate, may change over time.

**Table 3:** **Theoretical model* Grasping the lifeline ***

LOSING the	**GRASPING the**	HOLDING on to the
**LIFELINE**	**LIFELINE**	**LIFELINE**
**Lack of control**	**Taking control**	**In control**
Being mastered by others	Striving to be one’s own master	One’s own master
Having HC assistants not suited to needs	Selecting HC assistants	HC assistants suited to needs
**In unsafe hands**	**Seeking safe hands**	**In safe hands**
Feeling unsafe in care procedures	Instructing unskilled HC assistants	Safe in care procedures
Feeling unseen and unheard	Trying to be seen and heard	Seen and heard
**Insecure in the care system**	**Navigating the care system**	**Secure in the care system**
Lack of interest	Making own contacts	Good contact
Lack of connection	Coordinating own care	Connection

### Taking control

The patients tried to *take control* when cared for at home, despite their large care needs and dependency. Their dependency made them feel at risk of being mastered by others and having HC assistants not fitted to their needs*.* Their desire to participate in decisions that concerned themselves and their care was strong, which made them strive to be their own masters and to select their own HC assistants, to give a feeling of control (Table 3).

### Striving to be one’s own master

When striving to be their own master, patients felt they needed to fight for their rights: “They are here for me and not the other way around”*.* One patient believed she needed more help and strived hard to get her care planners to understand her needs.

"I will not stop fighting for my rights. Now I have two daytime HC assistants and one during the night. But I want two at all times (p)."

If the patient was unable to strive for control because of poor health a HC assistants could step in. One patient was unable to convey her desires verbally. “You need to interpret her correctly,” the HC assistant said about her*.*

### Being mastered by others

When patients lacked control they were not given the opportunity to make decisions and felt mastered by others. “Everyone gets to decide except me,” one patient said when HC assistants decided where to place the furniture in her own apartment. Another patient experienced being mastered by a health care professional.

"The physiotherapist decides everything and she always thinks she is right. I had a wheelchair I liked but she came and changed to another one, without asking me. This one I can’t use indoors and I want my old chair back. I told her “I’m the one with needs – not you!” (p)"

### One’s own master

When home care was the choice of the patient, the choice created a feeling of satisfaction: “To decide for yourself, to be able to live at home, nothing can compare with that!” one patient said. The HC assistant’s attitude to the patient’s right to decide was also important in terms of feeling one’s own master. “Yes, it is her apartment, she decides!” one HC assistant said.

### Selecting HC assistants

The patients were dependent on their HC assistants and thus wished to take part in the selection process. “I really want to participate in the selection of HC assistants. Not everybody fits in here!” one patient said. To know in advance which HC assistant was coming was important, and the patients objected strongly if the HC assistants were not selected to fit their needs. “I have learned to protest loudly if they try to send HC assistants I don’t like”. When she needed to leave the security of the home, one patient described how she tried actively to select the HC assistants she could trust.

"When I’m leaving the house for a doctor’s visit I want to select certain HC assistants to come along. (p)"

### Having HC assistants not fitted to their needs

A lack of satisfaction with their HC assistants created substantial concerns and worries for the patients. One patient said: “Some of them shouldn’t have come in the first place. They should work in something else!” Owing to a high turnover rate among HC assistants, the patients were faced constantly with new persons caring for them, which was a stressful experience. “I get new HC assistants all the time” said one patient, who had 17 HC assistants and new persons coming for introductory shifts several times a week. The temporary HC assistants created most concern. “It feels like they hire people right from the street,” one patient said.

### HC assistants fitted to their needs

Patients who were allowed to participate in the selection of HC assistants felt in control. One patient involved in the selection of new HC assistants said that she now had HC assistants she could trust. Another way of having control was the ability to decide who had the required skill when it came to difficult care situations, such as helping with showering or using hoists and other medical or technical equipment.

### Seeking safe hands

The patients tried to *seek safe hands* when cared for by HC assistants, because this rendered them at risk of feeling unsafe during care procedures as well as feeling unseen and unheard. In their striving to feel safe they instructed their unskilled HC assistants in care procedures and also tried to be seen and heard (Table 3).

### Instructing unskilled HC assistants

When striving to feel safe, the patients instructed new or unskilled HC assistants in care procedures. “I have shown the girls, in case something happens. So I have some control”, one patient said. One HC assistant noticed that when her patient got stressed or irritated she started saying: “do it like this, don’t do it like that!”

### Feeling unsafe in care procedures

The patients felt at risk of not having their care needs fulfilled because of *unskilled HC assistants*. One patient said, “They get it wrong many times. But I can’t do it myself so I have to live with it”. Lack of knowledge was also supported by one HC assistant: “I know nothing of my patients’ medical history. Where I worked before there was a file with information but I haven’t found one here.”

### Feeling safe during care procedures

The performance of care procedures by an experienced HC assistant with the skills to handle the medical and technical equipment correctly made the patient relaxed and safe.

"The HC assistant handles the hoist with experienced hands. For a moment the patient is hanging in the air before gradually being lowered down to the wheel chair. (obs.)"

"The HC assistant puts her hand on the patient’s chest in order to detect secretion in the airways. She then prepares the suction supplies and starts to remove the secretion from the tracheotomy. She is quiet and works efficiently. (obs.)"

### Trying to be seen and heard

Communication with the HC assistants was central to the patients as they strived to be seen and heard. As a consequence, language skills were an important issue. One patient, who had experienced misunderstandings during care, now demanded that the HC assistants spoke sufficient Swedish for them to understand each other, “so that things don’t go totally wrong”. Patients who could not move needed HC assistants to be close in order to communicate their needs. One patient said: *“*I do not dare to have the door closed, I need to hear them at all times,” and another said “When my nose is itching I have to call to get attention, so they come and scratch it for me – I am totally helpless!” The patient with impaired speech due to a tracheotomy was able to obtain the attention of the HC assistants by looking at them, and sometimes a glance was enough for the HC assistant to understand her needs.

### Feeling unseen and unheard

The observations revealed several situations in which the patients appeared to be unseen and unheard*.* For example, care situations were performed without the HC assistants engaging with or listening to the patient, which rendered her unseen by the HC assistants. During one observation, the patient lay completely naked in bed, with three persons in the room, while the HC assistants kept talking to each other. Another observation revealed a competing interest from the television.

"The TV is turned on, at a very high volume. The HC assistant manipulates the feeding tube with one hand and watches the TV at the same time. She changes TV-channel with the remote control using the other hand and keeps on watching. (obs.)"

Lack of communication skills in the HC assistant could create feelings of being unheard. When the HC assistant did not speak or understand Swedish (the patient’s language), the patient felt unsafe. One patient, in despair when her HC assistants did not understand the instructions on what to do, said: “If I tell them one thing and they do the total opposite, then something is wrong”.

### Seen and heard

The response of the HC assistants to the patients’ striving to be seen and heard seemed to depend on the experience and competence of the HC assistants. One way of showing patients that they were seen and heard, was through caring physical contact.

"“Come here darling.” The HC assistant touches the patient gently and turns her towards herself. The other HC assistant continues with the washing procedure. (obs.)"

Another way of showing patients that they were seen and heard was when the HC assistant, by knowing the patient’s wishes, could fulfill specific needs.

"When the washing procedure is done the HC assistant prepares and lights a cigarette and hands it to the patient. The HC assistant holds the cigarette. No words are spoken (obs.)"

When the patient could not verbalise her wishes, the strivings to be seen and heard were less obvious, thus demanding close attention from the HC assistant.

"You must learn when she does so and so with her eyes…. I do not know if it is correct or wrong, but I am almost always right with what she wants to say. (a)"

### Navigating the care system

The patients tried to *navigate the care system* when they felt insecure in a care system that was not fitted to their needs, i.e. they felt a lack of interest from their professional care providers as well as a lack of connection within the care system. To compensate for this lack, the patients tried to maintain their own contacts as well as coordinating their own care within the care system, sometimes with help from their HC assistants. The patients felt secure in the care system when they experienced good contact with the professional care providers as well as connection within the care system (Table 3).

### Making their own contacts

The patients established their own contacts when they experienced a lack of interest from their professional care providers. One patient was satisfied because she had established direct contact with the hospital instead of having to go through the primary care centre: “Nowadays I just call one of the doctors at the hospital and they help me”.

### Lack of interest

The patients experienced a lack of interest from professional care providers.

"Sometimes when I call the home care supervisors they say they can’t help me. I don’t think they can say that. They are there for me! (p)"

According to the patients and their HC assistants, few professional care providers made home visits and contacts were sparse. Sometimes, meetings that concerned the patients were held outside the patient’s home, which prevented them attending. Patients also expressed that they felt insecure when the routines did not work and they thought it was due to poor planning and lack of interest in their situation. One patient said: “They (the home care supervisors) didn’t have it under control and there were many things to complain about”. One patient felt that she was not important owing to un-kept promises.

"They promised me an outdoor wheelchair. But everything was postponed and I ended up sitting indoors all summer long. (p)"

The HC assistants of the patient who required a home ventilator thought that the health care professionals showed a lack of interest because they found the patient too complicated and did not want to learn more about her situation. The HC assistants said they had to beg for a home visit when the patient needed to be seen by the physician, because his office had no elevator, which made transport complicated. When the physician finally came, he stopped at the doorstep, totally startled when “he finally understood how complicated she (the patient) was,” one HC assistant said.

### Good contact

One patient experienced good contact with her home care supervisors following a reorganisation within her community. The improvements were evident to her HC assistants as well.

"When I first started here I came to a workplace extremely well organized with good routines. All things in the right spot. (a)."

Good contact with the health care professionals meant that patients became known within the care system. One patient had undergone surgery three years ago and now had her own medical chart at the local hospital. “She’s been living in this city with her large care needs and her home ventilator her entire adult life and they (the hospital) didn’t even know she existed!” one HC assistant claimed.

### Coordinating their own care

The patients coordinated their own care when they experienced a lack of collaboration within the care system. One patient coordinated the care herself and did not want to change that. “One professional care provider has nothing to do with the other,” she said. Another patient, with impaired verbal skills due to a tracheotomy, had to rely on her HC assistants and their ability to take over contacts and coordination. The HC assistants argued that, by coordinating care when there was no collaboration among professional care providers, they could solve small problems before they increased in magnitude and became dangerous, which prevented complications and even hospitalisation of the patient.

### Lack of connection within the care system

Patients as well as their HC assistants experienced a lack of connection within the care system, which resulted in no collaboration between the professional care providers.

"No, they do not speak with each other. They are on different planets, totally separated from each other. (p)"

For one patient, the lack of connection was the result of the district nurse and the physician coming from different primary care centres.

"The patient is still listed at Primary Care Centre A, where she used to live. There she has her primary physician. But her district nurse comes from Primary Care Centre B, where she lives now, so they cannot collaborate, as they need. (a)"

Being responsible for the safety of the patients, the HC assistant often had to turn to the professional care providers for advice and help. They often felt they had to step in and coordinate because of a lack of collaboration.

"As soon as she needs medical care we have to call several persons. They can’t even call or send a mail to each other (a)."

The lack of connection became particularly important when a patient was in need of *acute care* and could not possibly coordinate her own care but had to rely on the HC assistants. The HC assistants did not always know who to contact or experienced lack of interest when they contacted the health care professional they thought appropriate. Owing to unclear routines, the HC assistants felt unsupported when they needed assistance with the patient. During evenings and nights, when the regular primary care centre was closed, the HC assistants found it difficult to get the required assistance. “We called but no one answered,” one HC assistant said. Moreover, difficulties in obtaining help from the national emergency number (112) were described. When one patient needed acute care the HC assistants were startled when they called 112 and realised they could not expect immediate assistance. “We dialed 112 in order to get an ambulance. But we were told they did not have one to send at that time!” Given that they were responsible for a patient on a home ventilator, the lack of help was described as very stressful by the HC assistants.

"They said they wouldn’t take her in the ambulance because she needed to be put on the ventilator, and it had to be a special ambulance for that. (a)."

### Connection within the care system

When the patient experienced good contact with, as well as collaboration between, professional care providers in the care system she felt secure.

"I have never experienced any problems. It works smoothly with all the contacts: doctors, home health and social insurance office, you name it. (p)"

When there was collaboration, it usually resulted in good routines for acute care. For one patient, a routine to treat acute infections had made life much easier, with tests being taken at home and prescriptions called in by a doctor at the local hospital. Before the routine was established, she had to visit the emergency room frequently.

**Figure 1:**
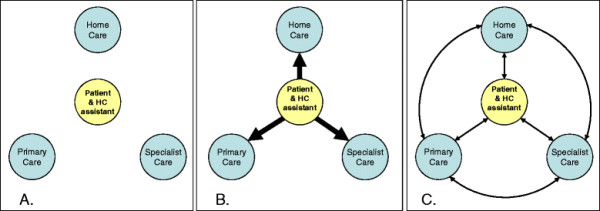
**The care system from the perspectives of the patients and their HC assistants. A**. *Insecure in the care system*. Lack of interest from professional care providers and lack of connection in the care system. **B**. *Navigating the care system*. Patients and/or HC assistants compensating for lack of interest and lack of connection. **C**. *Secure in the care system.* Good contact and connection in the care system.

To summarise, three variations on how patients and their HC assistants experienced the care system are shown in Figure 1: (A) *Insecure in the care system*, illustrated by an absence of connections between the patient/HC assistant and the professional care providers; (B) *Navigating the care system*, illustrated by the compensatory process when the patient/HC assistant maintained their contacts with the professional care providers and coordinated the care themselves; (C) S*ecure in the care system*, illustrated by existing connections between all actors involved.

## Discussion

The study described herein investigated the main concern of patients who needed 24-hour home care by HC assistants. Data from patient and HC assistant interviews were combined, together with observations of care situations. The initial findings were that patients who were in difficult life situations due to complex care needs but wished to stay at home were exposed to care systems not always fitted to their needs. The patients exposed states resulted in lack of control and unsafe care, which made them feel as if they were *losing their lifeline*. However, the patients compensated for their exposed states by using the core process of *grasping the lifeline*, as illustrated in the theoretical model. When striving for control, the patients tried to select their own HC assistants, instruct unskilled HC assistants in care procedures, and navigate the care system, sometimes with the help of a devoted HC assistant.

Despite the considerable care needs of these patients and their awareness of the risks of becoming exposed, they had chosen to stay at home with HC assistants, because the alternative was to live in a nursing home or in a hospital setting. Their strong desire to stay at home and to be in control is in agreement with previous studies on patient experience. Ballangrud *et al*[7] described chronically ill patients and their feeling of being in good health as long as they stayed in control. Several of the exposed states identified in this study, namely lack of coordination among health care professionals, lack of HC assistant competence and communication skills, and high turnover rates, have been emphasised earlier [12,16]. The contribution of the present study was the identification of compensatory processes to overcome these shortcomings.

When *compensating*, the patients were seen to be striving for control and safe care. The compensatory processes identified might be similar to descriptions in the literature of coping [35]. However, whereas coping is defined commonly as a reaction and effort to manage internal stressors [36], the compensatory processes seen in the present study were mainly actions taken as result of external flaws experienced in the care system. The compensatory processes were all related to patients’ strivings to make decisions and participate. Dreyer & Steffensen [6] found decision-making to be an important factor for patients experiencing life-changing situations, whereas Liley & Manthorpe [37] argued that both information and influence from family members are needed before decision-making. In the present study, the desire of patients to make decisions whenever possible and to participate in their own care is understood to represent their compensation for a care system that is not suited to their needs.

Trying to *select their own HC assistants* was determined to be an important compensatory process. The patients compensated for HC assistants who were not suited to their needs by trying to select certain HC assistants for specific situations and by controlling their work schedules to obtain certain competency when needed. When the patients could choose HC assistants they trusted and relied on, it made them feel safe. The importance of competent HC assistants to trust and rely on has been stressed in the literature, both from the perspective of patients and family [8] and from the perspective of the care provider [38]. In addition, all the patients mentioned it was stressful when they did not know who would come and care for them the next day, which echoes earlier studies that emphasise the importance of personnel continuity in home care [39].

The results of the present study emphasised the importance for the patient of the HC assistant’s competence in specific care situations. Instructing HC assistants in specific care procedures was another compensatory process used by patients when they felt unsafe in the hands of their HC assistants. This process could be understood as patients striving to maintain autonomy by self-care [11], but it could also be a sign of striving for safe care when HC assistants were not suited to their needs. The phenomenon was also seen in earlier studies that described patients with home ventilators teaching their HC assistants skills in basic hygiene as well as equipment safety [7,13]. Problems that occurred as a result of communication difficulties made the patient feel unsafe in the hands of the HC assistant because they could not use the compensatory process of instruction.

Patients in the present study navigated the system of care, with or without help from their HC assistants, either by making their own contacts with professional care providers and/or by coordinating the care themselves. By this means, they compensated for the experienced lack of interest from and lack of collaboration between professional care providers. Difficulties concerning joint responsibilities within Swedish organisations for home health care, which result in patients with the most complex needs being at the highest risk of neglect, have been described by the National Board of Health and Welfare [12,15].

In the present study, the patients and their HC assistants experienced a lack of collaboration between the home care supervisors and health care professionals from various settings, and regarded the groups as being on different “planets”. The lack of collaboration in the support of patients who need home care in Sweden is already known, although large regional differences exist [19]. The importance of good collaboration for successful home care is described well in earlier studies [38,40], which emphasise effective communication and consistent personnel as crucial factors for continuity. However, to our knowledge, the patients’ own navigating role while experiencing a care system not fitted to their needs has not been described previously. The finding emphasises the effort patients are prepared to undertake in order to be able to stay at home and live a life as close to normal as possible.

Patients in need of acute care experienced difficulties in receiving appropriate assistance from health care professionals. In these situations patients were not able themselves to use compensatory strategies but had to rely on their HC assistants. The HC assistants sometimes felt they had to beg for help, and on-call systems did not work as they were supposed to. When acute problems arose, the HC assistants were hampered by a lack of routines and support. Even calling 112 was unsuccessful on one occasion when they needed help with a patient on a home ventilator. This created a potentially unsafe care situation for the patient, as well as a stressful situation for the HC assistants. The importance of developing effective routines for acute care has been stressed by other authors [13,37,41]. Imasio & Yamauchi also emphasised the importance of establishing routines and instructions in the case of emergency situations in home settings [42].

Patients who suffered problems with breathing, either problems with equipment or signs of deteriorating breathing capacity, created particular stress for the HC assistants. Gysel & Higginson [43] found that patients with breathing problems created the utmost stress for their caregivers, when they needed to handle these problems in the home setting far away from professional support.

The results of the study highlight issues that are important for professional care providers within both home care and primary and specialist health care. By considering patients’ experiences and thoughts, as well as their use of compensatory processes, when planning and organising home care, the planned care might be more appropriate to and safe for the individual patient. However, patients’ use of compensatory processes might also be problematical and the patient perspective should be contrasted with the care perspective. The selection of HC assistants by patients is complex and involves several dilemmas concerning HC assistants’ working environment. These issues require more thorough exploration and research. Given that the present study focused on the patients, the perspectives of HC assistants on their own role within home care need to be investigated further.

### Credibility and limitations

The study was based on a small group of patients, but owing to the larger number of HC assistants that was included it has produced a great variety of interactional data. Sampling was not possible without the help of a supervisor at the local municipality, which might have influenced the selection of the four patients enrolled in the study. However, the disparities in the patients’ conditions and attitudes, as well as in the HC assistants’ attitudes and actions, suggest that any influence was not systematic. The lead author had professional experience of health care in a hospital setting but not in the home setting, which was an advantage in terms of having knowledge of the health care context. However, it might have been a disadvantage in relation to the HC assistants. An observer always has an influence on the observed situation [33]; in this case the presence of a registered nurse might have influenced the HC assistants to give a more positive representation of their part in the care than was typical. However, the authenticity of the situations is supported by the fact that both positive and less positive care situations were observed.

During an interview with the patient with impaired speaking abilities caused by a tracheotomy, the HC assistant interpreted words that the researcher could not understand fully, which could have altered the answers. However, the lead author has experience of working with patients with speech impairment and was able to combine whispering and body language from the patient with the HC assistant’s interpretation.

The lead author has worked in close collaboration with the other authors to enhance credibility [34,44], i.e. that the findings are believable and acceptable and that there is a good match between the researchers’ observations and the findings presented. A thorough and accurate description of methodology and results, illustrated with quotations and extracts from the material, provides the basis for credibility or fit [45]. Memos were written, collaborative analyses with researchers from other disciplines were undertaken, and the results were debated at several academic seminars. Rigour was ensured by transcribing all interviews immediately after the interview, in addition to writing memos, and by comparing new categories constantly with raw data and new data with established categories, during the analyses.

The theoretical model that resulted from the present study is a substantive theory applicable to the context from which it emerged, i.e. patients with complex care needs receiving 24-hour home care from HC assistants in a medium-sized Swedish city. Although grounded in empirical data, the model should be regarded as a set of proposals that might be applicable to similar situations and contexts.

## Conclusions

By combining interviews with patients and health care assistants (HC assistants) with field observations, the study yielded authentic data that provided a deeper understanding of patients’ experiences and thoughts when receiving 24-hour home care.

The results indicate that patients with complex care needs appreciate the opportunity to stay at home with HC assistants around the clock, despite the fact that the home care is not always suited ideally to their needs and there is a risk of unsafe care. We found that patients receiving 24-hour home care strive for control and safety by developing compensatory processes, for example selecting and instructing HC assistants and coordinating their care themselves.

The theoretical model, which is the main result of the study, illustrates some challenging areas for improvement within the organisation of home care, such as personnel continuity and competence, collaboration among care providers, and the development of routines for acute care. These issues require additional research that considers the perspective of HC assistants on their role as well as the patient perspective. The model may be used as a basis for reflection during the planning of care for individual patients as well as in discussions that concern home care in the future.

## Competing interests

The authors declare that they have no competing interests.

## Authors’ contributions

LS and IH designed the study. LS collected the data and drafted the manuscript. All authors have been involved in the data analysis and have read and approved the final version of the manuscript.

## Pre-publication history

The pre-publication history for this paper can be accessed here:

http://www.biomedcentral.com/1472-6955/11/9/prepub
